# Community-and proximate-level factors associated with perinatal mortality in Nigeria: evidence from a nationwide household survey

**DOI:** 10.1186/s12889-019-7151-0

**Published:** 2019-06-24

**Authors:** Osita K. Ezeh, Edward O. Uche-Nwachi, Uchechukwu D. Abada, Kingsley E. Agho

**Affiliations:** 1 School of Science and Health, Western Sydney University, Locked Bag 1797, Penrith, NSW 2571 Australia; 2 College of Medicine, Federal University, Ndufu-Alike, Ikwo, Ebonyi state Nigeria; 3 Department of Banking and Finance, Madonna University Nigeria, Okija Campus, Okija, Anambra state Nigeria

**Keywords:** Perinatal mortality, Proximate factors, Pregnancies, Nigeria, Newborn care

## Abstract

**Background:**

The perinatal mortality rate (PMR) in Nigeria rose by approximately 5% from 39 to 41 deaths per 1000 total births between 2008 and 2013, indicating a reversal in earlier gains. This study sought to identify factors associated with increased PMR.

**Methods:**

Nationally representative data including 31,121 pregnancies of 7 months or longer obtained from the 2013 Nigeria Demographic and Health Survey were used to investigate the community-, socio-economic-, proximate- and environmental-level factors related to perinatal mortality (PM). Generalized linear latent and mixed models with the logit link and binomial family that adjusted for clustering and sampling weights was employed for the analyses.

**Results:**

Babies born to obese women (adjusted odds ratio [aOR] = 1.46, 95% confidence interval [CI]: 1.13–1.89) and babies whose mothers perceived their body size after birth to be smaller than the average size (aOR = 1.92, 95% CI: 1.61–2.30) showed greater odds of PM. Babies delivered through caesarean section were more likely to die (aOR = 2.85, 95% CI: 2.02–4.02) than those born through vaginal delivery. Other factors that significantly increased PM included age of the women (≥40 years), living in rural areas, gender (being male) and a fourth or higher birth order with a birth interval ≤ 2 years.

**Conclusions:**

Newborn and maternal care interventions are needed, especially for rural communities, that aim at counselling women that are obese. Promoting well-timed caesarean delivery, Kangaroo mother care of small-for-gestational-age babies, child spacing, timely referral for ailing babies and adequate medical check-up for older pregnant women may substantially reduce PM in Nigeria.

## Background

Perinatal mortality (PM) includes foetal death from 22 weeks of gestation (stillbirth) and death within the first week after live birth (early neonatal death) [1]. PM reflects the effectiveness of healthcare provided to mothers and their babies during the antepartum, intrapartum and postpartum periods. It remains a significant global public health issue, particularly in developing countries, where approximately 98% of perinatal deaths occur [1]. A recent global estimate on childhood mortality suggested that approximately 4.8 million perinatal deaths occurred in 2009; of these deaths, 2.6 million were stillbirths while the remainder were early neonatal deaths [2]. These deaths are mostly linked to intrapartum-related stillbirths or newborn deaths; for example, nearly 50% of the 2.6 million stillbirths worldwide occurred in the intrapartum period [2, 3]. Perinatal deaths are preventable with optimal healthcare services, such as adequate receipt of antenatal care during pregnancy, skilled healthcare personnel assistance during labour and delivery, as well as quality care during the postpartum period.

Stillbirths and early neonatal deaths remain unacceptably high in Nigeria. Nigeria currently ranks second globally with nearly half a million stillbirths [4] and third with approximately a quarter million neonatal deaths (0–27 days) [5]. Every year in Nigeria, over 75% of neonatal deaths occur during the early neonatal period (0–6 days) [6]. The 2013 Nigeria Demographic and Health Survey (NDHS) report on childhood mortality indicated a reversal in gains made over previous years in reduction of perinatal mortality rates (PMRs) from 39 to 41 perinatal deaths per 1000 births between 2008 and 2013 [6], indicating a slight increase by 5.1%. Considering this statistic, the Federal Ministry of Health formulated and adopted the Nigeria Every Newborn Action Plan, whose core aims include a strategic plan to significantly reduce preventable newborn deaths and stillbirths in Nigeria by 2030 [7]. This policy initiative lacks comprehensive information on effective ways to treat the health risks experienced by mothers and newborns, such as antepartum bleeding, foetal growth retardation and preterm birth, particularly at the community level where about 66% of births occur at non-healthcare facilities [6, 7]. This is concerning because past studies have shown that antepartum bleeding [8] and foetal growth retardation elevate the risk of PM [9].

Few studies of PM have been conducted [10, 11] in Nigeria, but these studies were all hospital-based with small-scale surveys tailored to fit specific geopolitical populations. These studies indicated that low birth weight, maternal age, low number of antenatal visits, antepartum haemorrhage, prolonged labour, caesarean delivery, multiple gestation, unbooked pregnancies and birth asphyxia were associated with perinatal deaths. Limitations of these studies are that stillbirths and early neonatal deaths that occurred at non-healthcare facilities were not included and their findings may not be generalisable to the wider Nigerian population. Second, environmental factors were not considered in these studies despite the reported relationship between stillbirths and indoor air pollution in earlier studies [12, 13]. Prior to this study, there were no population-based studies assessing PM or population-attributable risk (PAR) estimates adjusted for independent risk factors related to PM in Nigeria.

Accordingly, this study used national household data, which captured every locality and ethnic group in Nigeria, to investigate the community-, socio-economic-, proximate- (or closest maternal, delivery and newborn characteristics) and environmental-level factors associated with PM. We also estimated the adjusted PAR proportions to provide the magnitude of the PM attributable to each significant risk factor in Nigeria. Estimates from this study will greatly assist health and economic policymakers in formulating cost-effective evidence-based national intervention programs to ensure that existing limited healthcare resources are used appropriately to scale down PM across the six geopolitical zones, including rural, metropolitan and urban areas in Nigeria.

## Methods

The 2013 NDHS cross-sectional household dataset publicly available online was used in this study. Information relating to pregnancies (stillbirths and live births) was detailed by eligible women aged between 15 and 49 years. Approximately 38,948 women were successfully interviewed during the 2013 NDHS survey. The statistical methodology used to report the number of pregnancies was described previously [7]. There were 31,121 pregnancies (weighted total) within the 5-year period (2008–2013).

In the study analysis, stillbirths (≥28 weeks gestation) and singleton early neonatal deaths (0–6 days) were combined and defined as PM. Information regarding perinatal deaths was restricted to the 5-year period to reduce recall errors of birth or death dates by mothers.

### Dependent variable

The dependent variable was PM defined as death of foetuses at or after 28 weeks of gestation to newborn first week of age. The dependent variable takes a binary form such that “PM” was observed as a “case” (1 = if death occurred between 28 weeks of gestation and first week of age) and “non-case” (0 = if death did not occur between 28 weeks of gestation and first week of age).

### Potential independent study factors

Independent variables used for the study analyses were based on previous studies on stillbirths and early neonatal mortality [10–12, 14, 15], especially in low- and middle-income countries. In addition, Moseley and Chen’s [16] framework of factors impacting child survival was also considered. The incorporated factors were adapted to the available data in the 2013 NDHS and these factors were categorized into four distinct level groups: i.e., community-, socio-economic-, proximate- and environmental-level factors.

The community-level factors comprised place of residence (classified as rural–urban residence) and geopolitical zone (classified as north-east, north-central, north-west, south-east, south-west and south-south).

The socio-economic-level factors included maternal marital status, education, work status and paternal education. Another socio-economic factor was the household wealth index, which estimated the economic strength of each of the households interviewed during the survey period. Households were classified as rich, middle and poor.

The proximate-level factors were maternal factors (body mass index (BMI), desire for pregnancy and age at birth); delivery factors (birth place, mode of delivery, and delivery assistance); and child factors (birth size, sex, birth interval and birth order). Delivery assistance herein refers to birth deliveries assisted by trained birth attendants (doctors, nurses, midwives) or untrained birth attendants (traditional birth attendants, relatives or other persons). Birth weight at birth was not used because almost 50% of the newborns were not weighed at the time of birth [6], but perceived newborn size at birth by mothers (classified as small or very small, and average or large) was used in place of birth weight. Environmental-level factors were also considered because previous epidemiological studies have suggested that the exposure of pregnant women to high levels of particulate air pollutants from soil or dust particles, solid fuel or tobacco smoke, during pregnancy is significantly associated with stillbirths [12, 17]. Consequently, cooking fuel (classified as non-solid fuel or solid fuel) was included in the analyses.

### Statistical analysis

The PMRs for all the potential coexistings were obtained. A multivariable Generalized linear latent and mixed models (GLLAMM) with the logit link and binomial family that adjusted for clustering and sampling weights was used to identify the associations between perinatal deaths and independent factors. In addition, crude estimates were also obtained for each of the independent factors.

A stage modelling approach similar to that of Dibley et al. [18] was adopted for the multivariable analyses. We entered each of the level factors (i.e., the community-, socio-economic-, proximate- and environmental-level factors) into the multivariable model one at a time to investigate their relationship with the study outcome, starting with community-level factors at 5% significance. This was followed by a manual elimination process. Variables with *p*-values < 0.05 were retained in the model (model 1). In the second stage of modelling, socio-economic-level factors were added to model 1 and significant variables with *p*-values < 0.05 were retained in the model (model 2). A similar process was followed in the third stage of modelling, i.e., proximate-level factors were assessed with the variables that were significantly related to the mortality outcome in model 2, and again those variables with *p*-values < 0.05 were retained in the model (model 3). In the final stage of modelling, environmental-level factors were entered into model 3, and only those variables with p-values < 0.05 were retained in the final model. All analyses were carried out with STATA software (v. 13.1; Stata Corp, College Station, TX, USA).

The total risk of perinatal deaths in the population between 2008 and 2013 attributable to each of the significant independent variables in the final multivariable model was measured by calculating PAR using the formula below:

$$ \mathrm{PAR}=\mathrm{pr}\ \left(\mathrm{aOR}-1\right)/\left(\mathrm{aOR}\right) $$

where pr is the weighted proportion of deaths during perinatal period (between 28 weeks of gestation and first 1 week of age) and aOR is the adjusted odds ratio for PM.

## Results

There was a weighted total of 1196 perinatal deaths comprising 396 third semester stillbirths (≥28 weeks of gestation) and 800 singleton early neonatal deaths (0–6 days) during the 5-year study period (2008–2013). A greater PMR was observed among newborn babies whose mothers were obese compared with those who had normal weight (PMR: 58 vs 37). Newborn babies delivered through caesarean section (CS) had a higher PMR than those who had a vaginal birth (PMR: 73 vs 25). Similarly, newborn babies perceived by their mothers as small or very small had a higher PMR than those of average or larger size (PMR: 46 vs 21) (Table 1).

**Table 1:** PMR with 95% CI for each category of community-, socio-economic-, proximate- and environmental-level variables in Nigeria, 2008–2013

Variables	Number of pregnancies	Perinataldeathsˇ	Rate (95% CI)
Community-level factors			
Geopolitical Zone			
North-Central	4197	129	31 (25–36)
North-East	5546	239	32 (38–48)
North-West	11,606	475	41 (37–45)
South-East	2718	95	35 (28–42)
South-West	2847	101	36 (29–43)
South-South	4208	158	38 (32–43)
Residence type^a^			
Urban	10,803	349	32 (29–36)
Rural	20,318	848	42 (39–45)
Socioeconomic-level factors			
Household wealth index^a^			
Rich	5198	176	34 (29–39)
Middle	11,303	403	36 (32–39)
Poor	14,621	617	42 (39–46)
Mother’s education^a^			
Secondary or higher	9796	338	35 (31–38)
Primary	5960	260	44 (38–49)
No education	15,366	599	39 (36–42)
Mother’s working status			
Working	19,963	749	38 (35–40)
Not working	9795	377	38 (35–42)
Mother’s marital status			
Currently married	29,803	1134	38 (36–40)
Formerly/never married	1319	63	47 (36–59)
Father’s education			
Secondary or higher	12,526	462	37 (33–40)
Primary	5709	226	40 (34–45)
No education	12,091	470	39 (35–42)
Proximate-level factors			
Mother’s age^a^			
< 20	1606	99	62 (49–74)
30–39	11,522	389	34 (30–37)
20–29	14,862	551	37 (34–40)
40–49	3132	158	50 (42–58)
Mother’s body mass index (MBMI)			
Normal (18.5 ≤ MBMI ≤24.9)	20,441	766	37 (35–40)
Underweight (MBMI > 18.5)	2592	83	32 (25–39)
Overweight (25 ≤ MBMI ≤29.9)	5408	202	37 (32–43)
Obese (MBMI ≥30)	2397	138	58 (48–67)
Wanted pregnancy at the time			
Wanted then	27,903	705	25 (23–27)
Wanted later	2009	42	21 (15–27)
No more	475	14	29 (14–45)
Sex			
Female	15,238	341	22 (20–25)
Male	15,488	460	30 (27–32)
Mother’s perceived baby size at birth			
Average or larger	25,719	532	21 (19–22)
Small or very small	4429	202	46 (39–52)
Birth rank and birth interval			
First child	6119	221	36 (31–41)
2nd or 3rd child, interval > 2	7237	137	19 (16–22)
2nd or 3rd child, interval ≤ 2	2766	92	33 (27–40)
4th or more child, interval > 2	10,781	202	19 (16–21)
4th or more child, interval ≤ 2	3823	150	39 (33–46)
Delivery assistance			
Healthcare professional	11,481	312	27 (24–30)
Non-healthcare professional	11,054	245	22 (19–25)
Mode of delivery			
Non-caesarean	29,819	747	25 (23–27)
Caesarean section^+^	577	42	73 (51–95)
Place of delivery			
Health facility	10,869	287	26 (23–29)
Home	19,537	470	24 (22–26)
Environmental-level factors			
Type of cooking fuel			
Non-solid fuel	5801	211	36 (31–41)
Solid fuel	24,978	958	38 (36–41)

NB: All perinatal mortality rate (PMR) estimates with 95% confidence interval (CI) were rounded to a whole number; ˇ Perinatal deaths, a combination of stillbirths and singleton newborn deaths; ~Rate, based on total number of deaths per 1000 (singleton live births + stillbirths); ^a^ The estimated PMR in the study differs from that reported by the 2013 Nigeria Demographic and Health Survey because we excluded multiple births; ^+^Caesarean section is detailed as elective and emergency caesarean in the survey

Multivariable analyses revealed that babies born to older mothers (age ≥ 40 years) had significant odds of perinatal deaths (adjusted odds ratio (aOR) = 1.35, 95% confidence interval [CI]: 1.06–1.73) compared with those born to mothers aged < 20 or 30–39 years. Babies whose birth size was perceived as small or smaller by their mothers were 1.92 times more likely to die during the perinatal period than those perceived as average or larger in size. Male babies (aOR = 1.45, 95% CI: 1.25–1.68) were also more likely to die during the perinatal period than their female counterparts, as were babies delivered through CS (aOR = 2.85, 95% CI: 2.02–4.02) (Table 2).

**Table 2:** Adjusted and unadjusted independent variables significantly associated with perinatal deaths in Nigeria, 2008–2013

Variables~	Unadjusted OR (95% CI)	Adjusted^a,b^ aOR (95% CI)
Community-level factors		
Residence type		
Urban	Ref	
Rural	1.27 (1.10–1.47)	1.33 (1.11–1.60)
Proximate-level factors		
Mother’s age		
30–39	Ref	Ref
< 20	1.92 (1.53–2.42)	1.27 (0.91–1.78)
20–29	1.08 (0.94–1.23)	0.96 (0.80–1.17)
40–49	1.44 (1.19–1.74)	1.35 (1.06–1.73)
Mother’s body mass index (MBMI)		
Normal (18.5 ≤ MBMI ≤24.9)	Ref	Ref
Underweight (MBMI > 18.5)	0.85 (0.68–1.07)	0.77 (0.58–1.02)
Overweight (25 ≤ MBMI ≤29.9)	1.03 (0.88–1.21)	1.12 (0.92–1.36)
Obese (MBMI ≥30)	1.43 (1.17–1.75)	1.46 (1.13–1.89)
Sex		
Female	Ref	Ref
Male	1.43 (1.24–1.65)	1.45 (1.25–1.68)
Mother’s perceived baby size at birth		
Average or larger	Ref	Ref
Small or very small	1.92 (1.61–2.29)	1.92 (1.61–2.30)
Birth rank and birth interval		
2nd or 3rd child, interval > 2	Ref	Ref
First child	1.95 (1.57–2.42)	1.81 (1.43–2.78)
2nd or 3rd child, interval ≤ 2	1.76 (1.34–2.31)	1.76 (1.34–2.31)
4th or more child, interval > 2	1.01 (0.81–1.26)	0.93 (0.73–1.19)
4th or more child, interval ≤ 2	2.05 (1.62–2.61)	1.86 (1.44–2.41)
Mode of delivery		
Non-caesarean	Ref	Ref
Caesarean section^c^	3.15 (2.25–4.40)	2.85 (2.02–4.02)

Variables that were associated with perinatal deaths; ^a^ Independent variables adjusted for: geopolitical zone, place of residence, wealth index, mother’s (marital status, education, age, body mass index, work status and desire for pregnancy), father’s education, child’s (sex, birth place, body size, mode of delivery, delivery assistance, birth order and birth interval, cooking fuel); CI, confidence interval; Ref, reference category. ^b^ Multiple births were not included in the analysis; aOR, adjusted odds ratio based on logistic regression; ^c^ Caesarean section is detailed as elective and emergency caesarean during the survey

Other significant factors that elevated perinatal deaths during the study period were babies born to mothers living in rural areas (aOR = 1.33, 95% CI: 1.11–1.60), babies of a fourth or higher birth order with a birth interval ≤ 2 years (aOR = 1.86, 95% CI: 1.44–2.41), babies of first birth order (aOR = 1.81, 95% CI: 1.43–2.78) and babies of a second or third birth order with a birth interval ≤ 2 years (aOR = 1.76, 95% CI: 1.34–2.31). In addition, babies born to obese mothers were more likely to die (aOR = 1.46, 95% CI: 1.13–1.89) compared with those having low BMI (Table 2).

Between 2008 and 2013, approximately 18% of perinatal deaths in Nigeria were attributed to babies whose mothers reside in rural areas (PAR = 0.176, 95% CI: 0.07–0.28). During the same period, approximately 4% of perinatal deaths were attributed to babies whose mother’s BMI (MBMI) was ≥30 kg/m^2^ (PAR = 0.037, 95% CI: 0.010–0.070). Similarly, around 12% of perinatal deaths were attributed to male babies (PAR = 0.119, 95% CI: 0.070–0.170) (Table 3).

**Table 3:** Estimated PAR for each of the variables significantly related to perinatal mortality in Nigeria, 2008–2013

Variable	n*Ψ	aOR*	PAR (95% CI)
Community-level factors			
Residence type			
Urban	29.2	Ref	
Rural	70.8	1.33	0.176 (0.065–0.282)
Proximate-level factors			
Mother’s age			
30–39	32.5	Ref	
< 20	8.3	1.27	
20–29	46.1	0.94	
40–49	13.2	1.35	0.034 (0.006–0.068)
Mother’s body mass index (MBMI)			
Normal (18.5 ≤ MBMI ≤24.9)	64.4	Ref	
Underweight (MBMI > 18.5)	7	0.77	
Overweight (25 ≤ MBMI ≤29.9)	17	1.12	
Obese (MBMI ≥30)	11.6	1.46	0.037 (0.010–0.0702)
Birth rank and birth interval			
2 or 3 child, interval > 2	11.4	Ref	
First child	18.4	1.81	0.082 (0.048–0.137)
2 or 3 child, interval ≤ 2	7.7	1.76	0.033 (0.016–0.055)
4 or more child, interval > 2	16.8	0.94	
4 or more child, interval ≤ 2	12.5	1.86	0.058 (0.032–0.088)
Child sex			
Female	28.5	Ref	
Male	38.4	1.45	0.119 (0.070–0.170)
Mother’s perceived baby size			
Average or larger	44.5	Ref	
Small or very small	16.9	1.92	0.081 (0.053–0.114)
Mode of delivery			
Non-caesarean	62.4	Ref	
Caesarean section^+^	3.5	2.85	0.023 (0.013–0.038)

*Weighted proportion of perinatal deaths

Ψ variable proportion did not add up to 100 due to missing values

*The adjusted model included place of residence; geopolitical zone; household wealth index; mother’s (marital status, education, working status, age, body mass index, desire for pregnancy); father’s education; child sex; place of birth; delivery assistance; mode of delivery; child’s body size at birth; birth order and birth interval and cooking fuel

*aOR* adjusted odds ratio, *PAR* population-attributable risk, *Ref* reference category

## Discussion

The risk of perinatal death in Nigeria between 2008 and 2013 was significantly elevated by a range of risk factors, including babies born to older women (≥40 years of age), residing in rural areas, male gender, mother being obese (MBMI ≥30 kg/m^2^), having small or smaller birth size, birth through CS and having a fourth or higher birth order with a short birth interval ≤ 2 years after adjusting for necessary coexisting risk factors, including environmental-level factors.

The limitations of the current study are: (1) It is possible that over- or under-estimation may have affected PM risk estimates identified in this study because characteristics such neonatal jaundice, gestational age, sepsis or congenital anomalies, which were earlier found to be significantly related to early neonatal deaths in various hospital-based studies [10, 11] were not available for investigation. It is also possible that included characteristic such as perceived newborn size at birth by mothers may have impacted PM estimates because the rationale or criteria used was unclear. The perceived newborn size at birth by mothers could be limited by mother’s prior knowledge and experience of newborn. (2) Information on medical risk factors related to mothers during pregnancy, such as diabetes mellitus, hypertension and genitourinary tract infection [19, 20] previously found to be significantly associated with PM were lacking in the 2013 NDHS. (3) It has been argued that using early-pregnancy weight and height measurements yield more accurate BMI [21]; however, the current study assumed that MBMI at the time of survey was the same as that prior to the index birth, which may have biased our findings. (4) The number of newborn deaths and stillbirths may have been under-reported because only surviving mothers were interviewed. Additionally, mothers may have erroneously reported the gestational age when they had a stillbirth.

Notwithstanding these aforementioned weaknesses, this study used a nationally representative sample compared with earlier hospital-based studies in Nigeria [10, 11]. More importantly, this study suggested country-wide evidence on primary key factors associated with PM, which will enable healthcare policymakers to formulate uniform intervention programs to improve child survival across all six geopolitical zones and the Federal Capital Territory of Nigeria. In addition, we explored more data on many possible coexisting risk factors related to PM, including environmental-level factors, for sufficient statistical control.

Babies born to obese mothers had a 1.46 times greater risk of dying in the perinatal period than those having normal MBMI (18.5 ≤ MBMI ≤24.9). Similar findings were reported in earlier studies conducted in both developing and developed countries Nigeria [22], sub-Saharan Africa [23], Russia [24] and Sweden [25]. An increased likelihood of perinatal deaths for babies born to obese mothers may be attributed to undiagnosed diabetes, gestational diabetes or prediabetic hyperglycaemia, hypertension and large-for-gestational-age birth, which were previously found to be associated with foetal or newborn deaths [26]. This is supported by observational studies on maternal obese and pregnancy outcome in Nigeria, which found that diabetes mellitus, birth asphyxia, hypertensive disorders and macrosomia were significant medical risk factors associated with pregnant obese mothers in Nigeria [27]. Another possible reason for the elevated perinatal deaths among obese mothers may be because they are ineffective at recognising decreased foetal movement, which often leads to foetal demise compared with underweight or normal weight mothers [28]. The impact of obesity remains a crucial and amendable risk factor for PM. Effective maternal weight loss or control in obese women during pregnancy has been reported to reduce gestational diabetes, large-for-gestational-age birth and neonatal mortality [29].

The odds of perinatal deaths were greater in older mothers aged 40 years or higher compared with younger mothers. This finding is in contrast with earlier studies in Russia and Bangladesh [19, 30], which showed a statistically insignificant relationship between older mothers and perinatal deaths. A plausible explanation for these differing outcomes may be attributed to different definitions of PM, different populations, adjusted for different risk factors and the maternal age limits used. Nevertheless, other reports from both developing and developed countries also indicated that older mothers had a significantly elevated risk of perinatal death [31, 32]. The significantly increased perinatal deaths associated with older Nigeria mothers could be related to medical risk factors, such as antepartum haemorrhage, hypertension, operative vaginal delivery and gestational diabetes, which were previously reported to be associated with advanced maternal age [8, 26].

Babies perceived as small size after birth by their mothers remain a significant public health problem in Nigeria. A recent Nigerian population-based study suggested that nearly 30,000 newborn deaths in their first week of life were attributable to small-sized babies [15]. The current study provides further evidence that smaller-than-average babies is a strong predictor of perinatal death in Nigeria, which is consistent with findings from previous studies [15, 33]. A plausible reason for the increased risk of perinatal deaths among the small-sized babies in Nigeria may be attributed to use of herbal medicines not prescribed [34] and poor diet intake during pregnancy often impact the foetus.

Furthermore, studies in low- and middle-income countries [35, 36] have reported that babies born to mothers residing in rural residences are more likely to experience PM compared with those in urban areas, and this study reaffirms the strong effects of rural residence on perinatal deaths. This may be due to inadequate access to healthcare facilities and maternal healthcare services. Most of the well-equipped hospitals and healthcare centres in Nigeria are typically found in urban and metropolitan areas, leading to a lower likelihood of perinatal deaths in urban areas. The risk of perinatal death was significantly greater for male babies than for female babies. This may be attributed to biological factors [37], such as late development of foetal lung in the first 7 days of life [38], resulting in more frequent respiratory diseases in male babies than in female babies.

Babies delivered by CS exhibited an almost threefold risk of perinatal death compared with vaginally born babies, which contradicts a previous study conducted in Swaziland [39]. However, our result is consistent with a similar study performed in Mexico [40]. An increased risk of CS may be linked to negative perceptions (e.g., fear, aversion) to CS [41] among pregnant women in Nigeria, which often leads to late referral to healthcare facilities for emergency CS after experiencing life-threatening complications at home or elsewhere. We also observed that babies of high birth order (fourth or higher) born with shorter birth intervals (≤2 years) had greater odds of dying during the perinatal period. This outcome is in line with an earlier study conducted in Kenya [42], which may be partly attributed to economic resource competition among infants and maternal depletion syndrome [43].

To accelerate progress towards reducing prenatal mortality in Nigeria by 2030, public health interventions are needed at the community and individual level. At the community level intervention, screening programmes during pregnancy should be encouraged among older mothers especially those who live in rural areas, At the individual level intervention, educating mothers on kangaroo mother care approach on small-sized or low birth weight newborns including those delievered by caesarean section. However, to achieve these goals, the political, economic and social obligation of the three tiers of government (Federal, State, and Local Goverment Area) are very important to substantially reduce perinatal deaths.

## Conclusion

This finding suggests that newborn care interventions are urgently needed, at the community and individual level that aim to educate mothers on the benefits of weight loss or control, kangaroo mother care of small-sized babies, child spacing, timely referral for unwell babies, planned or elective CS, and promote regular medical monitoring of older pregnant mothers. These interventions will remarkably improve the rate of PM in Nigeria.

## Data Availability

The data set supporting the conclusions of this article is available at https://dhsprogram.com/data/dataset/Nigeria_Standard-DHS_2013.cfm?flag=1 and access to this data set is granted by the MEASURE DHS/ICF International or from the corresponding author.

## Abbreviations

aOR: Adjusted odd ratio
BMI: Body mass index
CI: Confidence interval
CS: Caesarean section
DHS: Demographic and Heath Survey
ICF: Inner City Fund
Kg/m: Kilogram/Metre
MBMI: Mother body mass index
MD: Maryland
NDHS: Nigeria Demographic and Heath Survey
PAR: Population attributable risk
PM: Perinatal mortality
PMR: Perinatal mortality rate
pr: Proportion
TX: Texas
USA: United States of America
VS: Versus
